# Federalism and representation: Evidence from state abortion laws in the aftermath of Dobbs vs. Jackson women’s health organization

**DOI:** 10.1093/pnasnexus/pgaf125

**Published:** 2025-05-13

**Authors:** Gabor Simonovits, David Doherty, Alexander Bor

**Affiliations:** Department of Political Science, Central European University, Vienna 1100, Austria; Rajk College for Advanced Studies, Budapest 1085, Hungary; Department of Political Behavior, Institute for Political Science, HUN REN, Budapest 1097, Hungary; Department of Political Science, Loyola University Chicago, Chicago, IL 60611, USA; Democracy Institute, Central European University, Budapest 1051, Hungary

**Keywords:** abortion, decentralization, representation, Supreme Court

## Abstract

Supporters of devolution argue that local policies better reflect citizen preferences than “one size fits all” policies enacted at the federal level. To test this claim, we leverage the sudden devolution of abortion policy-making that resulted from the *Dobbs* decision. Using multilevel regression with poststratification, we estimate the latest gestational age at which the average resident of each state believes abortion should be permitted and compare these estimates to state policies before and after the *Dobbs* ruling. We demonstrate that policies prior to *Dobbs* were more liberal than the average constituent’s preference in every state. In the wake of *Dobbs*, although this nationwide liberal bias evaporated, absolute distance between public preferences and policy was essentially unchanged. Instead of bringing policies closer to preferences, devolution allowed more liberal states to maintain policies that were “too liberal” for their average resident and opened the door for conservative states to leapfrog the preferences of their constituents.

**Significance Statement**

This study examines how the US Supreme Court’s Dobbs decision, which devolved abortion regulation to states, affected policy alignment with public opinion. We compare state abortion policies to residents’ preferences before and after Dobbs, testing a key claim of federalism: that local governance better reflects citizen views. We find that devolution did not improve overall policy-preference alignment. Pre-Dobbs, policies were more permissive than average preferences nationwide. Post-Dobbs, while this liberal bias disappeared, conservative states often enacted stricter laws than residents preferred, while liberal states maintained permissive policies. These findings challenge assumptions about federalism’s ability to enhance representation on contentious issues, offering crucial insights for US governance debates.”

## Introduction

Debates about the appropriate balance of power between the federal and state governments have been a feature of the political landscape in the United States since its founding. Over time, political power has drifted toward the national level. Supporters of this trend argue that some policy matters require nationwide coordination and that some rights are too fundamental to be left to the discretion of individual states. Those who support devolving power to the states argue that state policies tend to more closely reflect the interests and preferences of residents of each state, improving policy representation in the country as a whole.

Assessing the extent to which devolving power to the states might affect substantive representation is difficult. A great deal of research has examined the relationship between either statewide policy preferences and state policies or nationwide preferences and national policy outcomes (see Ref. (1), for a review). However, no work that we are aware of has empirically considered the counterfactual of whether policy representation in the United States would be improved if one could “flip a switch” and allow states to make policy on a matter where they had previously been constrained by national policy. This is, in part, because changes in the balance of power in the federalist system tend to be gradual, multifaceted, and play out over decades. This challenge is compounded by the fact that estimating the distance between public preferences and policy is often difficult because measures of public opinion are rarely directly comparable to policy outcomes.

We overcome these issues by exploiting the Supreme Court’s 2022 decision in *Dobbs v. Jackson Women’s Health Organization*, which overturned 50 years of precedent that had barred states from limiting access to abortion during the early stages of pregnancy. This ruling created a readily identifiable, nationwide structural break in state-level policy-makers’ ability to respond to public sentiment on a key dimension of a salient issue. We take advantage of an open-ended survey question that asked respondents what they believed should be the latest point when a women should be allowed to have an abortion in their state (absent circumstances—e.g. threats to the health of the woman—where many states make exceptions to gestational age thresholds). Thus, our approach differs from measurement other scholars have used in that, instead of estimating whether the public supports or opposes a given policy proposal (2), we seek to estimate the distance between policy and the average resident’s ideal point in each state. We leverage multilevel regression with poststratification (MRP) to estimate the maximum gestational age at which the average resident of each state believed abortion should be permitted. We compare these estimates to state policy in each state just prior to *Dobbs* being argued and policy 2 years later, following that structural break in federalist arrangements regarding abortion policy. As illustrated below, a striking number of states enacted new abortion policies during this crucial period, demonstrating the salience of this issue and urgency with which policy-makers took advantage of their newfound freedom to legislate. We report estimates, weighting by state population, that can answer the question: did devolving power to the states improve policy representation in the country as a whole?

Our strategy addresses two challenges that have stymied researchers’ ability to assess the effects of devolution on substantive representation. First, we take advantage of a rare, sudden shift in rules regarding which level of government has discretion to make policy on a highly salient, “easy” issue (3). Second, our measures allow us to make direct comparisons between public preferences regarding one facet of abortion policy—the maximum gestational age at which abortion should be permitted—and the policies that bind the public in each state.

Our evidence suggests that, at least on this dimension of this issue, the *Dobbs* ruling led to a vastly stronger *relationship* between state-level public opinion and policy outcomes. However, changes in the wake of the *Dobbs* ruling, on average, failed to bring policy closer to the public’s ideal points. Instead, the steeper relationship between public preferences and policy outcomes is a product of policies that were more liberal than the average resident’s preference remaining in place in some states, and many conservative states “leapfrogging” over average residents’ preferences and enacting policies that were notably more conservative than the public would prefer (4). Taken together, the findings suggest mixed conclusions about the extent to which devolution is likely to enhance representation. The gestational age “floor,” first established by the US Supreme Court in *Roe*, inhibited variation in policies across states and appears to have been more liberal than many Americans—and, more narrowly, many state publics—would prefer. However, rather than making policies more in line with public preferences, withdrawing those limits sharpened policy polarization across states.

## Devolution and the quality of representation

Most democratic theorists posit that some degree of congruence between public preferences and policy-makers’ behavior is an essential feature of a functioning democracy (5–7). Many observers argue that delegating policy-making to legislators at lower levels enhances representation (8–10). This view is based on the assumption that elected officials acquire high quality information about constituents’ policy preferences and prioritize catering to these preferences out of reelection concerns. Thus, the benefits of decentralization relative to national-level policy-making purportedly come from local politicians having an accurate picture of their (narrower) constituencies’ preferences and flexibility to tailor policies to those preferences. If these assumptions hold, we would expect state-wide policies in a conservative (liberal) state to more closely match the preferences of the conservative (liberal) residents of that state than national policies. Ultimately, this would lead to improved policy representation in the country as a whole.

In practice, however, there are reasons to doubt that this stylized account of the representation relationship reflects the realities of the policy-making process. A rich literature has identified several factors that work against the representation of constituent preferences. First, political elites appear to harbor surprisingly inaccurate perceptions of their constituents’ preferences, including on salient issues (11–13). Thus, even if legislators wanted to represent the policy preferences of their constituents, they may fail to do so because they lack quality information about what those preferences are. In addition, even if elected officials were aware of the preferences of their constituency at large, instead of catering to them, incumbents may discount those opinions as ill-informed or unlikely to be acted upon (14, 15) and instead respond to their party’s base (16, 17), more affluent citizens (18, 19), and organized interest groups (20).

The appeal of devolution, thus depends on the degree to which these problems are exacerbated at the state (as opposed to national) level of policy-making. There is reason to think they are. First, accessing accurate information about constituent preferences is likely to prove more difficult at the state level (21). Second, voters tend to have less information about state political dynamics than national dynamics (22), making it more difficult to hold elected officials accountable (23). Furthermore, although the rate of uncontested US Congressional races has declined in recent decades, the rate of uncontested state legislative races has increased (24) due to increased partisan sorting (25) and strategic redistricting plans (26). State legislators serving heavily conservative or liberal districts where the opposing party declines to even field a challenger may have little incentive to respond to average voters in their state, rather than their primary voters. Additionally, the fact that state legislative campaigns cost notably less than congressional campaigns may make it easier for organized interests and other well-resourced actors to wield influence over state policy-makers.

To summarize, theories of democracy often posit that electoral incentives tend to produce democratic responsiveness. However, in practice, an array of forces may interfere with this connection. This interference may be particularly pronounced at the level of state legislators who lack access to rigorous evidence about constituents’ preferences and may feel insulated from electoral consequences because they (reasonably) believe that few constituents are sufficiently informed to hold them accountable when policy outcomes diverge from their preferences.

Against the backdrop of these theoretical and methodological threads a long line of scholarship has attempted to evaluate how well variation in policies across American states represent public preferences. This literature has produced mixed normative conclusions. Some scholars have interpreted the evidence of *policy responsiveness*—a correlation between measures of mass preferences and policy outcomes across states—as painting a “reassuring portrait of statehouse democracy” (27). In contrast, others have identified “a striking democratic deficit” (2) by demonstrating a great deal of *policy incongruence*—measured as the mismatch between specific policies and majority opinion within states (2), or *policy bias* defined as the ideological distance between policies and average preferences (28).

While to our knowledge, no studies have considered how *devolution itself* shapes representation, the implication of which evaluative standard to use is hard to overstate. If one uses responsiveness to assess the quality of representation, decentralization is likely to be superior, as a national, one-size-fits-all policy (or, in the case of the *Roe* framework, a national policy that substantially restricts state policy-making discretion) clearly cannot respond freely to differences in preferences across states. However, through the lens of congruence or bias, policies enacted by states could fare worse compared to a single national policy if state policy-makers are ill-informed about constituents’ preferences or feel freer to pursue ideologically extreme policies (29, 30).

## Conceptualizing preferences, policies, and representation

We explore representation in the context of abortion laws and we focus on one key aspect of this contested policy: the latest point in a pregnancy (post-last menstrual period [LMP]) when abortion is permitted, absent an extenuating circumstances like health complications. One advantage of relying on this aspect of abortion regulation is that it allows the mapping of an otherwise complex policy on a simple cardinal scale (29) and, thus, direct comparisons of preferences and policies within states (31). Additionally, gestational age thresholds are arguably the most broadly consequential dimension of this policy space. Exceptions to gestational age thresholds are normatively important. However, the circumstances meriting exceptions under various state laws typically pertain to a small number of patients. For example, by some estimates abortions stemming from rape account for only 1% of abortions (32). The vast majority of abortions happen early in pregnancy for reasons tied to financial circumstances, relationship difficulties, and other life priorities not covered by policy exceptions. Furthermore, details regarding how exceptions are implemented in practice can be complex. For example, many states require women to report a rape to law enforcement—a requirement that may effectively preclude many women in these circumstances from seeking abortion. Similarly, doctors may be asked to make judgments about when health complications are serious enough to provide an abortion without running afoul of state law and may be averse to risking legal penalties if government officials disagree with their judgment.

This said, we recognize the limitations of our approach. For example, although we can estimate how close state policy is to average preferences among residents of a state, the policy consequences of various gestational age thresholds are asymmetric in ways that are difficult to operationalize. Because most abortions occur early in pregnancy, policy changes in the conservative direction affect more women. Only 3% of abortions occur in the 15–20 weeks gestational age window; 15% occur between 10 and 15 weeks (33). In a state where residents, on average, would prefer a gestational age threshold of 15 weeks, a policy that set a threshold at 10 weeks would *prohibit* five times more abortions that residents think should be permitted than a 20 week threshold would *permit* in contravention to public preferences. From this perspective, because post-*Dobbs* changes to gestational age thresholds were overwhelmingly changes in the conservative direction, our estimates may be a “best case” estimate of policy congruence. On the other hand, it may be that people view preventing one abortion at 20 weeks as more important than ensuring access to five abortions at 10 weeks.

As with any work that tries to assess the quality of political representation, we cannot fully address these complexities. Instead we acknowledge them and note that we believe that our approach is superior to available alternatives. The key benefit is that we are able to measure public preferences and state policies on the same scale and assess not only the *correlation* between state preferences and policies but also *congruence*. Ultimately, this positions us to answer our core research question: did the degree of policy representation realized *nationwide* improve following *Dobbs*?^a^

## Data and methods

### Measurement

A key step in our analysis is to estimate average policy preferences in each US state regarding the latest point in a pregnancy (post-LMP) when abortion should be permitted. We measure this “ideal policy” relying on an open-ended survey question—i.e. asking respondents to give their response on a numeric scale, rather than choosing between different policies (29). Echoing prevailing rhetoric regarding gestational thresholds, we asked survey respondents: excluding cases where the woman was the victim of rape or incest, where the health of the woman is endangered, or where there is a risk of serious defects in the fetus, how many weeks into a typical 40-week pregnancy should be the latest point when a woman should be allowed to have an abortion in their state.^b^ Our data are based on a nationally representative sample of 1,570 Americans in NORC’s AmeriSpeak Panel fielded in April 2023 complemented with 5,271 additional respondents relying on two Lucid omnibus surveys (both fielded in July 2023). We report sample demographics in the Supplementary material (Tables S1 and S2). We obtained informed consent from all survey participants and they were compensated for participation in the study. Our study did not include deception—and is unlikely to have caused discomfort in respondents. All research activities for this article received IRB approval from Central European University.

The average respondent’s gestational threshold is at 16 weeks. The median respondent is at 12 weeks. Twenty-three percent of respondents indicated that abortion should never be allowed (0 week), and 18% that it should always be allowed (40 weeks). The distributions of responses across the two sources and three samples are remarkably similar (see Fig. S1 in the Supplementary material). An array of analyses give us confidence in the validity of this measure (see Figs. S2–S6 and Tables S3, S4, and S12 in the Supplementary material). First, our data capture well-documented partisan and demographic differences in abortion policy preferences. Second, we further compare our measure with data from the ANES 2022 Pilot study that included questions about which trimesters should abortions be allowed under various circumstances. These comparisons reinforce confidence in the face validity of our results. Third, the face validity of our approach is also supported by a comparison of our measure with available state polls.^c^

Fourth, we also presented respondents with a series of profiles of hypothetical women and asked whether they thought “it should be possible” for the person described “to obtain a legal abortion in these circumstances?”^d^ These vignettes varied characteristics of the women including her age, racial identity, family income, marital status, existing number of children, and the reason for the abortion, including failure to use birth control; birth control was used, but failed; no reason was offered. The experiment also varied the gestational age (in weeks). We demonstrate the potent relationship between gestational age and support for permitting abortion. We also illustrate the clear relationship between patterns of response to these vignettes and responses to our open-ended question.^e^

Finally, we conducted a follow-up survey, again relying on Lucid fielded in November 2024 (n=2,683). Here, we also measured “open-ended” gestational age threshold preferences in the case of exceptional circumstances and probed the sensitivity of the responses to the provision of information about the pregnancy. We randomly assigned some respondents to a condition where these items were prefaced with information to contextualize fetal development throughout pregnancy. We found that including contextual information about viability and other developmental touchstones common in public debates did not change either the central tendency or the distribution of responses. Moreover, we found that preferences about gestational age limits are much more liberal in the case of exceptional cases, providing further evidence that our more granular measure of abortion preferences replicates findings based on more traditional approaches. We report these findings in detail in the Supplementary material (see Table S11, Fig. S12).

### Modeling and aggregation

To estimate state-level preferences, we rely on MRP (34). Specifically, we model abortion preference as a function of demographics (age, sex, education, and race), place of residence (state and region) as well as two state-level smoothers: Donald Trump’s two-party vote share in the 2016 Presidential election, and an abortion preference index from the 2022 Cooperative Election Study (CES) survey. Because our outcome variable is censored between 0 and 40, we use ordered beta regressions (35) relying on the ordbetareg package in R. Details about the model specification are provided in the Supplementary material (Fig. S7). As in other applications of MRP, this approach has the advantage of both correcting for the fact that our samples are not representative at the level of states and, by relying on partial-pooling, borrowing information from the full sample to help with inferences for states with smaller populations, where sample sizes are correspondingly smaller.

Finally, we use poststratification to obtain measures of abortion preferences in each state not only for the post-Dobbs period when our survey was fielded but also for the year *prior* to the Dobbs decision. To do so, we exploit the fact that the Cooperative Election Study (CES) asked the questions forming our state-level abortion preference index in both periods. These binary policy questions are not useful for quantifying policy responsiveness because none of the questions ask about gestational age limits; preferences and policies are not measured on the same scale. However, our multilevel regression demonstrates the CES abortion index is strongly predictive of gestational age preferences at the state-level (see Table S8 and Fig. S6 in the Supplementary material). For the post-Dobbs preference estimates, we follow standard poststratification procedure and predict the gestational age limit preference for each census cell based on the 2022 abortion index values (which were also used to fit the model). To estimate pre-Dobbs preferences, we predict each census cell using the 2020 CES abortion index values. As our survey data are limited to a single year, we are unable to estimate the year-to-year variability in the state-level correlation between CES-index and gestational age preferences. This issue likely makes our pre-Dobbs estimates overconfident. Yet, CES data and recent scholarship (36) show that abortion attitudes changed little between 2020 and 2022 and cannot account for the patterns we report below (see Fig. S8 in the Supplementary material). We aggregate from census cells to average state-level preferences using the 2022 and 2019 population weights from the American Community Surveys, for the post-Dobbs and pre-Dobbs period respectively (accessed via IPUMS USA, (37)).

### State policies

We compare the resulting sets of estimates of average state-level preferences to state policies just prior to 2021 December 1 (when the *Dobbs* case was argued) and policies as of 2023 November 30.^f^ In this 2-year window, 22 states changed their rules regarding the maximum gestational age at which abortion is permitted. In some cases, these changes were precipitated by “trigger laws” that state legislatures enacted anticipating the eventual overturning of *Roe*. In others, the state legislature moved rapidly in the wake of *Dobbs* to revise abortion laws. As we discuss below, abortion laws in some states are in flux as of this writing.

## Results

**Fig. 1.**
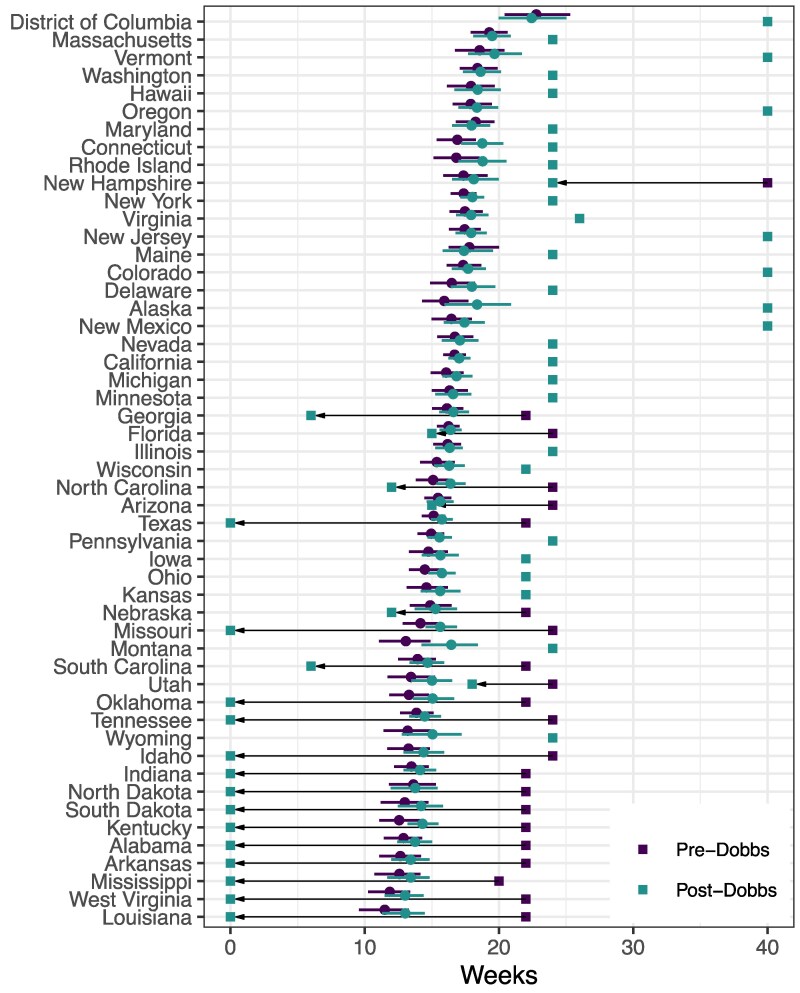
Abortion policies and preferences around Dobbs. Crosses denote state-level abortion policy pre-Dobbs (blue) and post-Dobbs (purple). Arrows highlight changes where they took place. Grey points denote the mean estimate of policy preference. Error bars denote 90% credible intervals.

Figure 1 plots our estimates of state preferences both before and after the Dobbs decision (solid dots with 90% credible intervals) and contrast these estimates with abortion laws regarding maximal gestational age just prior to oral arguments in *Dobbs* and as of November 30, 2023. In Table 1, we report key estimates of interest, weighting by state populations. The results indicate that, before *Dobbs* the gestational age thresholds across states were higher than the average residents’ preferences by, on average, 9 weeks (Liberal Bias row). Because thresholds were higher than public preferences in every state, this average Liberal Bias is identical to our estimate of the average absolute difference between policy and average preferences across states (Absolute Bias).

**Table 1.** Policy responsiveness.

	Pre-Dobbs	Post-Dobbs	Difference
State Policy	24.62	17.35	− 7.27
State Preference	15.81	16.46	0.65
	(14.65, 17.00)	(15.35, 17.58)	( 0.24, 1.03)
Liberal Bias	8.81	0.89	− 7.92
	( 7.62, 9.98)	(−0.23, 2.00)	(−8.31, −7.51)
Absolute Bias	8.81	9.20	0.38
	( 7.62, 9.98)	( 8.08, 10.30)	(−0.71, 1.50)
Responsiveness	1.16	6.07	4.91
	(0.57, 1.75)	(4.75, 7.39)	(3.53, 6.28)

All estimates are state averages, weighted by state population size. Uncertainty estimates in brackets denote 90% credible intervals. Detailed regression model estimates on responsiveness are reported in Supplementary material (Tables S5–S7).

Figure 1 also shows that the devolution of policy decisions resulting from the Supreme Court’s repeal of *Roe* only led to changes that lowered gestational age thresholds. No state *raised* its gestational age threshold following *Dobbs*. Our weighted average indicates that, these change almost entirely eliminated the Liberal Bias across states (Post-Dobbs column of Table 1). However, the reduction in average Liberal Bias masks a striking pattern. In most states that changed policy, although gestational age limits were “too liberal” for the average resident prior to *Dobbs*, new laws—many of which either outright banned abortion or prohibited it after six weeks (a point at which many women do not know they are pregnant)—were far more conservative than the average resident’s preference.

Consider Tennessee. Prior to *Dobbs*, abortion was permitted up until 24 weeks—about 10 weeks later than the average Tennessee resident’s preference for an approximately 14 week threshold. This 10 week bias was *exacerbated* by the change in Tennessee law that entirely banned abortion (unless the physical health of the woman was threatened). A similar pattern is apparent in many of the states in Fig. 1—particularly the more conservative states in the bottom portion of the figure. The end result is that, although devolution appears to have all but eliminated *directional* (here, liberal) bias in states’ gestational age restrictions, absolute bias was virtually unchanged (see Absolute Bias row in Table 1). A situation where almost all state policies tended to be “too liberal” was replaced by a new policy regime where policies remained “too liberal” in some states but were replaced by policies that were “too conservative” in others.^g^

**Fig. 2.**
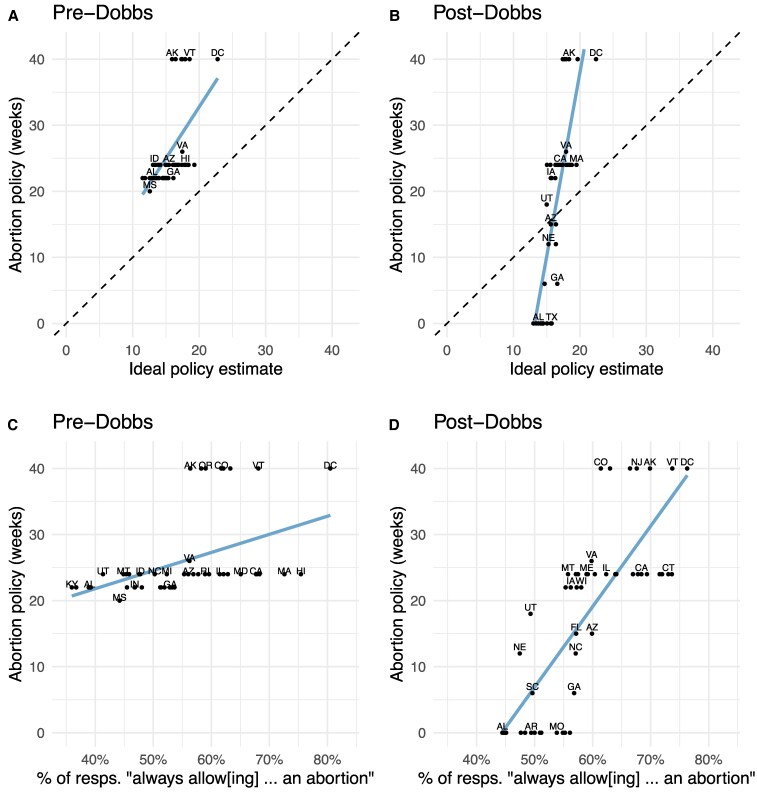
Policy responsiveness and bias in abortion laws. A and B) MRP-based ideal policy estimates against abortion policies in each state, pre- and post-Dobbs, respectively are displayed. C and D) The proportion of respondents in the Cooperative Election Survey (CES) in each state agreeing with the survey item “Always allow a women to obtain an abortion as a matter of choice” against the same ideal policy estimates is displayed. All points denote states. The blue lines denote ordinary least squares (OLS) regression lines.

The patterns that emerge in our analysis underscore the value of our methodological approach. As shown in Fig. 2A and B, the strength of the relationship between average preferences and state policy also increased dramatically from a slope of 1.16 to 6.07 (slopes reported in Responsiveness row of Table 1). This type of strengthened relationship could be identified with an array of measures of the public’s abortion attitudes (38, 39). We illustrate this in Fig. 2C and D, where we replicate our analyses instead drawing on data from the 2020 and 2022 CES surveys and using a more conventional measure to estimate state preference—the percentage of respondents that support the proposal to “always allow a woman to obtain an abortion as a matter of choice” (we report similar results based on other CES items in the Supplementary material, see Figs. S9 and S10).

We see a dramatic strengthening of the relationship between this alternative measure of state-level preferences and state policies. It would be tempting to read these results as indicating improved representation. However, as noted above, even though this conventional approach can demonstrate increasing *responsiveness* to state preferences, it cannot speak to whether this responsiveness reflects a reduction in policy *biases* or if, instead, *Dobbs* opened the door to *hyper-responsiveness* and sharpened policy polarization. In contrast, by placing public preferences and policy on a comparable scale, our approach allowed us to identify and quantify the substantial mismatch between preferences and policies both before and after *Dobbs*.

## Conclusion

The evidence presented here speaks to fundamental, long-standing debates regarding the extent to which bringing policy-making “closer to the people” enhances democracy. It is the first effort, to our knowledge, to directly assess how devolving policy-making power to the state-level affects substantive policy representation. Our findings indicate that devolution erased a broad, directional policy bias on this salient issue and increased the strength of the relationship between state preferences and state policy. However, to date it has failed to deliver on a key promise made by advocates of devolution: laws that more closely reflect the preferences of those bound to obey them.

Like all research, our evidence has limitations. The first is that we only examine the relationship between public attitudes and one dimension of abortion policy. Thus, we cannot speak to congruence between public opinion and the entirety of states’ abortion policy regimes. Another limitation is, discussed above, is that we cannot account for the possibility of asymmetries in this policy domain. Our analysis assumes that liberal and conservative biases are equivalent. It is not clear that this is the case on this policy. Given that the vast majority of abortions occur before eight weeks gestational age (40), laws that set very low gestational age thresholds or ban abortion outright affect a huge share of women who may seek abortion services. Policies that set the threshold higher than the public lead to a much smaller number of abortion outcomes that the public opposes. Other asymmetries may be rooted in a pattern where those who prefer more conservative abortion policies have more (or less) intense preferences than those who support more liberal policies.

An additional limitation is that states’ abortions policies are (and will likely always be) in flux. For example, before *Dobbs*, thirteen states passed “trigger laws” that would go into effect if *Roe* was overturned. Prior to *Dobbs*, state legislators may have viewed these as a way to cater to antabortion minorities without fear of legislative backlash. This said, nine of these laws were enacted after Justice Kavanaugh was confirmed and it was clear that *Roe* was at risk, a timeline that casts some doubt on the notion that legislators did not expect these laws to go into effect (41). Other states had pre-*Roe* laws on the book that had not been repealed. In the coming years, legislators may amend these pre-*Dobbs* laws to better reflect statewide sentiment. However, the massive amount of abortion legislation enacted during the period we examine here suggests that failure to do so thus far is not simply a matter of legislators being reluctant to make policy hastily.

Over time, some state publics may be able to bring policy into closer alignment with their preferences via direct ballot initiatives. For example, in 2024, Missouri voters used ballot initiative to override the state’s outright ban on abortion, though our estimates suggest that the viability (24 week) threshold specified in that advocate-crafted initiative may have “overshot” public preferences in that state. It is important to note that, in addition to the fact that many states do not have a referendum or initiative process, this avenue for enhancing substantive representation is typically not central to devolution advocates’ arguments. Instead, those arguments posit enhanced representation provided by democratically elected representatives. Prevailing dynamics on this issue suggest that, rather than being eager to let the voters have their say, democratically elected state legislators at times actively attempt to derail this process. For example, in Ohio—where Republicans enjoyed a commanding majority in both chambers of the legislature—Republican legislators led an effort to raise the threshold for amending the state constitution by referendum in the hopes of increasing the chances that a subsequent referendum to enshrine abortion rights in the state constitution would fail. In Florida, legislators passed a law changing a policy that largely banned abortion after 15 weeks—a threshold quite close to our estimate of preferences in the state—to prohibit abortion after 6 weeks. In November 2024, voters considered a referendum to permit abortion up through viability. Although a majority supported the referendum it fell short of the 60% threshold required to pass.

Our study takes advantage of a sudden shift in how policy-making power on an issue is distributed in the United States. Thus, our evidence speaks to whether the “treatment” embedded in the Supreme Court’s *Dobbs* ruling brought policy closer, on average, to public preferences. We cannot speak to the mechanics of why some states moved policy closer to their residents’ preferences or what reforms might enhance state-level policy responsiveness. Instead we show that devolving policy-making power to the states on this salient issue reduced directional policy bias and yielded an stronger estimated relationship between statewide preferences and state policies. However, it failed to improve the extent to which policies mirror the preferences of those bound to obey them. This is notable given the distinctive features of the abortion issue. Legislators could easily access information about public opinion on abortion in their states and the flurry of legislative activity in the wake of *Dobbs* suggests that legislators viewed the issue as likely to be something constituents cared about and were paying attention to. Thus, this would seem to be an issue where delegating authority to state-level representatives—rather than unelected national jurists—would be particularly likely to enhance substantive representation. Our estimates suggest that it did not.

## Supplementary Material

pgaf125_Supplementary_Data

## Data Availability

We share all data and computer code necessary to reproduce our analyses in a public repository, https://osf.io/gcz53/.

## Notes

a We emphasize that this question is about the system, rather than about why some states opted to revise policies and others did not or whether policy representation changed specifically in states with policies that, pre-Dobbs, were constrained by the floor set by the Roe framework. Thus, we aggregate policy bias across all states, regardless of whether they changed policies in the wake of Dobbs or their pre-Dobbs policies.

b Because pregnancy is counted from the first day of a person’s last menstrual period, this means a person is not actually pregnant for the first 2 weeks and few women are aware that they are pregnant at 4 weeks. Additionally, very few abortions are performed after 24 weeks (42). Thus, we replicate our analysis, top- and bottom-coding responses to restrict this measure to range between 4 and 24 weeks. The findings are similar to those we report below (see Table S9, Fig. S11 in the Supplementary material).

c We thank an anonymous reviewer for this suggestion.

d These questions were adapted from Doherty (43) and included in our NORC and first Lucid surveys; 3715 respondents evaluated a total of 14,410 hypothetical women.

e We attempted to use the experimental data to obtain alternative estimates of state-level preferences but the estimates were quite imprecise. Thus, we only use these data to validate our open-ended preference measure at the individual level

f State policies are from the Kaiser Family Foundation ((44), pre-Dobbs policies from Internet Wayback Machine version of page as of December 4, 2021; post-Dobbs policies updated 2023 November 2). For states that specify “fetal viability” thresholds, we use 24 weeks; we use 40 weeks for states with no clear viability threshold. Because the 6-week gestational threshold specified in the Texas Heartbeat Act (2021 September 1) was inconsistent with the *Roe* framework, we use Texas’ prior 22-week threshold as the pre-*Dobbs* policy in that state. New Hampshire enacted a law specifying a 24 week gestational age threshold on 2022 January 1. Prior to this, state law did not specify a gestational age limit. However, unlike the Texas law, this state law was not designed to challenge *Roe*. Thus, we code New Hampshire as having a 40-week threshold pre-*Dobbs* and 24 post-*Dobbs*. Note that this coding choice suggests that *Dobbs* increased congruence between public preferences and policy in New Hampshire, even though the policy change was not directly tied to that ruling. We demonstrate that our results are robust to excluding New Hampshire from the analyses in the Supplementary material (Table S10).

g Our data regarding preferences about gestational age limits in the case of exceptional circumstances—when the woman’s health is endangered, fetal abnormalities and pregnancies resulting from rape or incest shows that the public supports permitting abortion notably later in pregnancy under these circumstances. Post-*Dobbs*, many states do not provide for these exceptions at all. Our approach does not position us to measure the conservative biases that were likely introduced by these new state laws.
