# Supplementary material for: Federalism and representation: Evidence from state abortion laws in the aftermath of Dobbs vs. Jackson women’s health organization
Source: PNAS Nexus. 2025 May 13;4(5):pgaf125. doi: 10.1093/pnasnexus/pgaf125 (PMC12070389; doi:10.1093/pnasnexus/pgaf125)
Supplement: pgaf125_Supplementary_Data [file pgaf125_supplementary_data.pdf]

Federalism and Representation: Evidence from State Abortion Laws in the Aftermath of Dobbs vs. Jackson Women's Health Organization
Supplementary Material

Gabor Simonovits[*] CEU
David Doherty [†] LUC
Alexander Bor[‡] CEU

April 15, 2025

[*]Corresponding Author, (email: simonovitsg@ceu.edu). Associate Professor, Department of Political Science, Central European University, Vienna, Austria; Co-Director, Rajk College for Advanced Studies, Budapest, Hungary; Senior Researcher, HUN REN, Budapest, Hungary

[†]Professor, Department of Political Science, Loyola University Chicago, Chicago, IL, USA

[‡]Post-doctoral researcher, Democracy Institute, Central European University, Budapest, Hungary

Contents

A Survey data collection **2**

A.1 Demographics 3

A.2 Question wording 4

A.2.1 Open-ended question 4

A.2.2 Conjoint experiment 4

A.2.3 Benchmarking experiment 6

A.2.4 Additional survey items 6

A.3 Measurement validation 7

A.3.1 Group differences 7

A.3.2 American National Election Study 10

A.3.3 Experimental measurement approach 13

B Modeling and validation **14**

C Responsiveness estimates **16**

D CES and abortion policies **18**

E Top- and bottom-coding gestational age **22**

F Excluding New Hampshire **24**

G Benchmarking experiment **25**

H MRP estimates versus state polls **29**

A Survey data collection

We fielded the following four surveys during 2023:

1. NORC's AmeriSpeak Panel. N = 1,570. Data collection April, 2023. Contained both the open-ended question on preferred maximum gestational age for abortion as well as the conjoint experiment.

2. Lucid A. N = 2,202. Data collection in July, 2023 with identical materials to the NORC survey.

3. Lucid B. N = 3,069. Data collection in July, 2023. Contained only the open-ended question.

4. Lucid 2024 N=2683 Data collection in November, 2024 (the days before the elections).

Lucid Theorem is a leading survey aggregator in the US, offering online samples resembling the population in terms of demographics. Validation studies show that the estimates from Lucid closely follow those obtained by high-quality face-to-face sample, e.g. by the American National Election Study.[1] Lucid data has been also used in combination with MRP to study state-level public opinion.[2] Moreover, a recent study has demonstrated that Lucid respondents' views on abortion appear to mirror closely to margins found in probability samples.[3]

[1]Coppock, A., & McClellan, O. A. (2019). Validating the demographic, political, psychological, and experimental results obtained from a new source of online survey respondents. Research & Politics, 6(1), 2053168018822174.

[2]Simonovits, G., & Bor, A. (2023). Stability and change in the opinion–policy relationship: Evidence from minimum wage laws. Research & Politics, 10(3), 20531680231188262.

[3]Doherty, D. (2022). What Can Conjoint Experiments Tell Us about Americans' Abortion Attitudes?. American Politics Research, 50(2), 147-156.

A.1 Demographics

Table S1: Sample demographics. Cell entries are the (marginal) proportion of respondents within the given demographic category in each individual survey, and the total sample.

	NORC	LUCIDa	LUCIDb	Total
18-29	0.13	0.21	0.22	0.20
30-44	0.28	0.30	0.30	0.29
45-64	0.32	0.32	0.32	0.32
65+	0.27	0.17	0.16	0.19
Black	0.10	0.11	0.11	0.11
Hispanic	0.17	0.13	0.13	0.14
White	0.73	0.76	0.76	0.75
No High School	0.04	0.04	0.04	0.04
High School	0.20	0.26	0.26	0.24
Some College	0.41	0.22	0.22	0.26
College	0.21	0.32	0.33	0.30
Post Graduate	0.15	0.17	0.16	0.16
Men	0.49	0.49	0.49	0.49
Women	0.51	0.51	0.51	0.51

Table S2: Sample demographics 2024 follow up

	Lucid2024
18-29	0.12
30-44	0.29
45_64	0.39
65+	0.20
Black	0.12
Hispanic	0.03
White	0.85
No High School	0.03
High School	0.22
Some College	0.23
College	0.38
Post Graduate	0.13
Men	0.47
Women	0.53

A.2 Question wording

We rely on various approaches to measure preferences regarding the latest point in pregnancy when respondents support the legality of abortion.

A.2.1 Open-ended question

We asked the following question to measure Open-ended Maximum Gestational Age preferences (included in the first three surveys):

> Now we would like to ask you about abortion law in your state. In your opinion what number of weeks should be the LATEST point when a women should be allowed to have an abortion in your state? As a reminder, a typical full term pregnancy lasts 40 weeks. If you think women should not be allowed to have an abortion select 0. If you think women should be allowed to have an abortion at any point in a pregnancy, select 40. Otherwise select from the options in between. When you think about your answer, *do NOT consider circumstances where the woman was the victim of rape or incest, where the health of the woman is endangered, or where there is a risk of serious defects in the fetus.*

Figure S1 displays the distribution of our open-ended focal dependent variable across our three samples, demonstrating very high similarity, increasing confidence in the reliability of our data.

A.2.2 Conjoint experiment

In the experimental approach to estimate abortion gestational age preferences, we ran a standard conjoint experiment with the attributes displayed in Table S3. This set of questions were included in the the NORC survey and the Lucid A survey. We displayed one profile at a time, and five profiles in total for each respondent. Respondents were asked "Do you think it should be possible for NAME to obtain a legal abortion in these circumstances?". The response option were "Yes, should be possible", and "No, should not be possible".

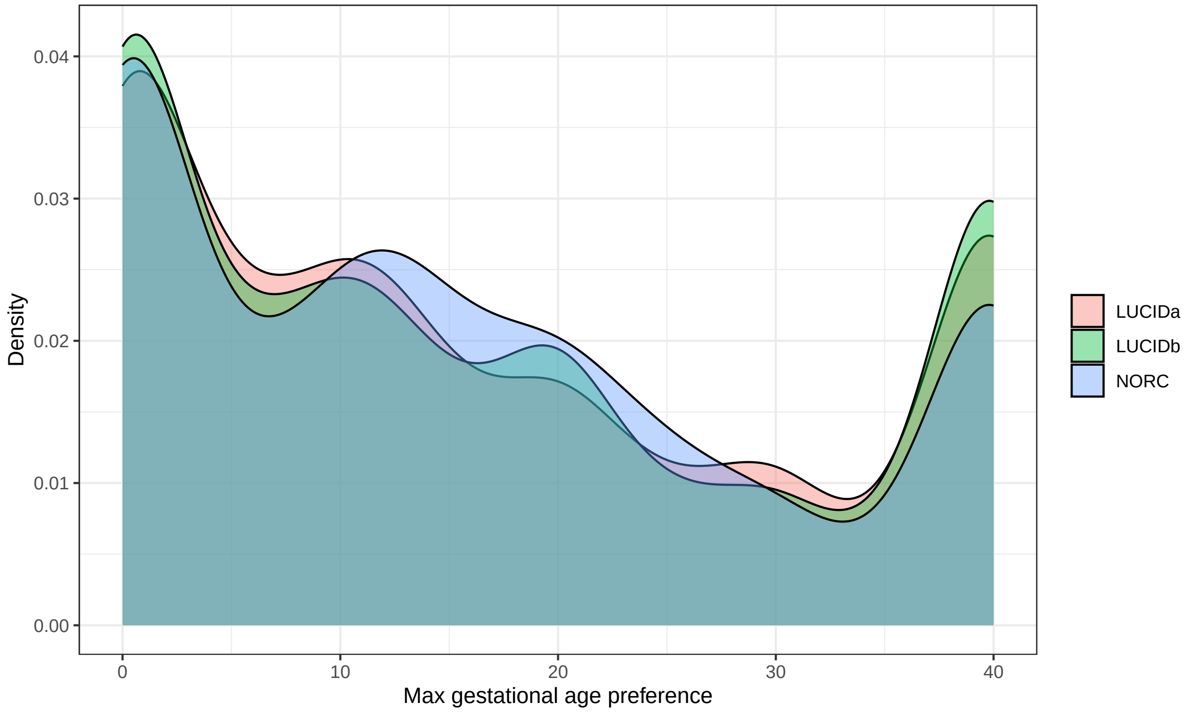

Figure S1: Distribution of maximum gestational age preference across the 3 surveys we fielded. The distributions are very similar across the two panels and three waves of data collection.

Table S3: Attributes and levels in the conjoint experiment

**Attributes**	**Levels**
Name (62)	e.g. Ebony Washington, Dolores Sanchez, Sarah Miller
Woman's age (37)	14–50
Weeks into pregnancy (full term = 40 weeks) (33)	4–36
Reason for abortion (3)	Did not use birth control, Used birth control but it failed, No specific reason provided
Annual family income (100)	$6,000 - $325,000
Marital status (2)	Married, Not married
Number of children woman currently has (10)	0, 0, 0, 0, 1, 1, 2, 2, 3, 4

Note: Numbers in parentheses denote the number of levels.

A.2.3 Benchmarking experiment

Finally, in our follow-up survey we included a version of the open-ended question with a subset of the respondents also exposed to 4 more benchmarks commonly mentioned in debates about abortion. The box below displays our question wording with the treatment highlighted in **bold**. The order of the first three items were randomized with the option "none of these circumstances apply" always coming last. 75% of our sample saw the version with the benchmark and the remaining 25% got the control.

> Now we would like to ask you about the abortion laws in your state. In your opinion, what number of weeks should be the LATEST point when a woman should be allowed to have an abortion in your state?
> As a reminder, a typical full-term pregnancy lasts 40 weeks. **The heart starts beating around week 5-6. The fetus starts to make active movements around week 14-16. If born prematurely, a baby in the US has around 50% chance of survival at week 24, and 95% chance from week 28.**
> If you think women should not be allowed to have an abortion please enter 0, below. If you think women should be allowed to have an abortion at any point of the pregnancy, please enter 40 below.
> Please consider each of the following circumstances and enter a number in between 0 and 40.
>
> - The health of the woman is endangered:
> - There is a risk of serious birth defects in the fetus:
> - The woman was victim of rape or incest:
> - None of these circumstances apply:

A.2.4 Additional survey items

In addition to there three focal items, we also relied on demographic profile variables on age, gender, educational attainment, ethnicity, partisanship and state of residence, as provided by the survey vendor – i.e. these survey questions were not included in our survey.

A.3 Measurement validation

We validate our measure of gestational age preferences at both the individual and aggregate level. First, we demonstrate that heterogeneity in gestational age limits in our data replicates well-known partisan and demographic differences in abortion policy preferences. Second, we demonstrate substantial overlap between our gestational age preferences and a measure included in the 2022 ANES pilot survey. Finally, we contrast our open-ended survey measure with a unique experimental approach to measuring gestational age limit preferences.

A.3.1 Group differences

First, we map group differences in gestational age limits. Specifically, Figure S2 displays average preference for age × gender caregories, education levels and racial categories. To ensure consistency, we use our main Bayesian multilevel ordinal beta regression model for our predictions. Replicating prior results, we see that younger and better educated respondents have more liberal preferences, although the differences are small. Surprisingly, we find that in our data and in this model, men are about 1.5 weeks more *liberal* then women within each age group.

We also explore partisan differences at the individual level. Because partisan identity is not part of our MRP model we do not have partisan identity data in the second 2023 Lucid survey data shared with us. The analyses below are therefore limited to 3,772 respondents. Table S4 and Figure S3 replicate well-known partisan differences in abortion policy preferences. Democrats are much more liberal (mean = 21.6 weeks (95%CI 21-22), median = 20 weeks) than Independents (mean = 15.6 weeks (95%CI 15-16), median = 12 weeks) or Republicans (mean = 10.4 weeks (95%CI 10-11), median = 6 weeks).

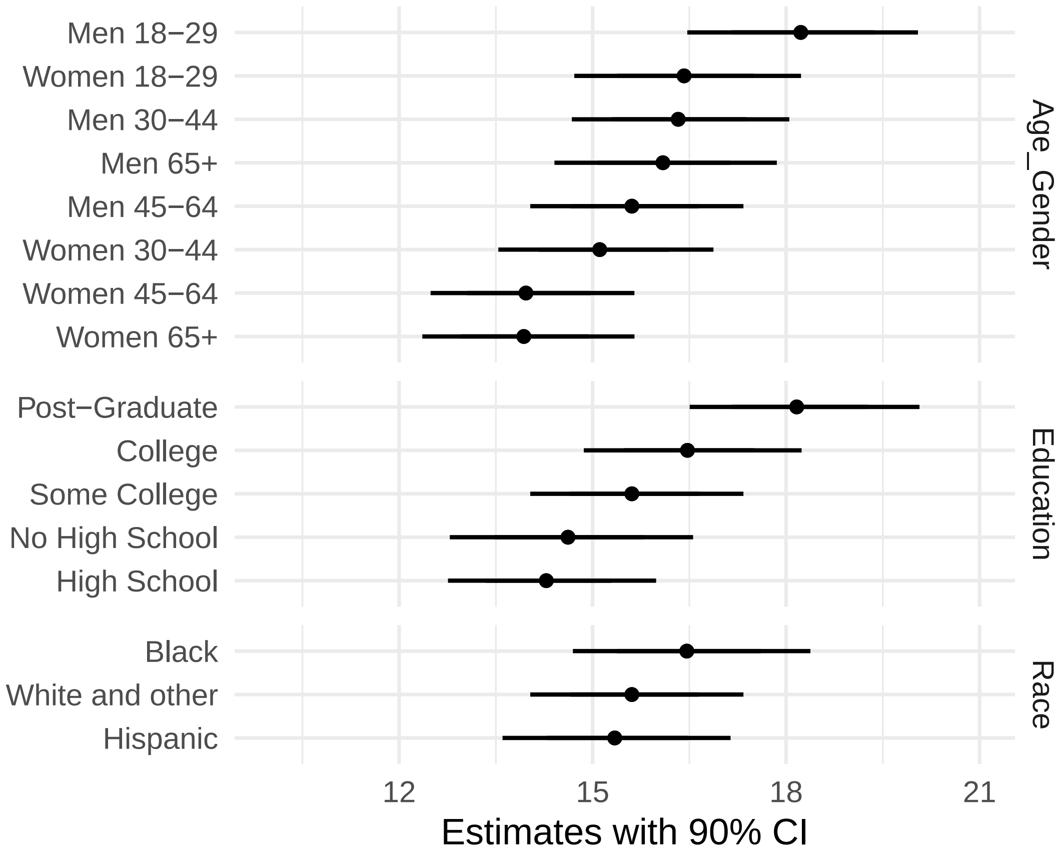

Figure S2: Demographic differences in average gestational age limits. Estimates are based on our our main multilevel Bayesian regression. When predicting preferences for a given demographic variable's subgroups, we fix all the other parameters to a neutral middle category – the state of Nebraska, to Whites, to men aged 45-64, and to some college.

Table S4: Mapping partisan differences in gestational age preferences

	Dependent variable:
	Gestational age limit preference
Democrats	21.6***
	(20.9, 22.2)
Independents	15.6***
	(14.6, 16.5)
Republicans	10.4***
	(9.7, 11.1)
Observations	3,772
Adjusted R^2	0.6
Residual Std. Error	13.5 (df = 3769)
Note:	*p<0.1; **p<0.05; ***p<0.01

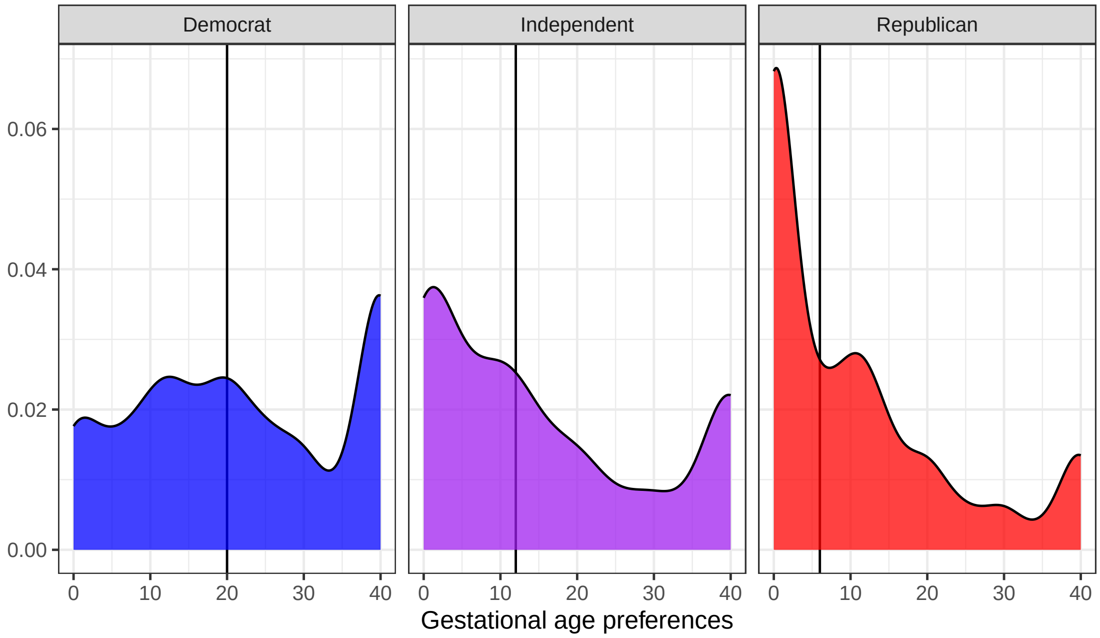

Figure S3: Partisan differences in the gestational age limit preferences. Vertical lines denote the median preference within each subgroup. Leaning independents are lumped together with partisan respondents.

A.3.2 American National Election Study

The 2022 American National Election Survey (ANES) pilot included the questions below with regards to respondents' gestational age limit preferences. Although these questions are less granular than our week-based measure, they allow a comparison between the two approaches.

For each of the following situations, please indicate if you think abortion should never be legal, if it should be legal only in the first trimester, legal in the first and second trimesters, or legal in the first, second and third trimesters.

Situations:

1. If the pregnancy is unwanted for any reason
2. If a serious birth defect is detected
3. If the pregnancy was the result of rape or incest
4. If the pregnancy puts the woman's life at risk.

Response options:

1. Never
2. First trimester only
3. First and second trimesters
4. First, second, and third trimesters

We transform our own measure by distinguishing between the following four categories of respondents:

1. Never - Answered 0 weeks
2. First trimester - Answer between 1 and 12 weeks (inclusive)
3. Second trimester - Answer between 13 and 27 weeks (inclusive)
4. Third trimester - Answer above or equal to 28 weeks

Figures S4 and S5 display the distribution of categories contrasting our own measure with ANES Pilot data. Most importantly, we find that the two distributions are consistently similar – we ought not to worry that our respondents give random, nonsensical answers. Besides, we find that ANES find somewhat more conservative distribution with more respondents saying never than in our more granular question. It is notable that these differences are largest in

the two questions with most conservative attitudes (any reason + birth defects). A plausible explanation for this tendency is that some respondents whose ideal point is above 0 but below 12 weeks feel closer with the ANES "never" option than stating that abortion should be legal in the (whole) first trimester. Again, this reinforces the importance of relying on a sufficiently granular measure to estimate policy congruence.

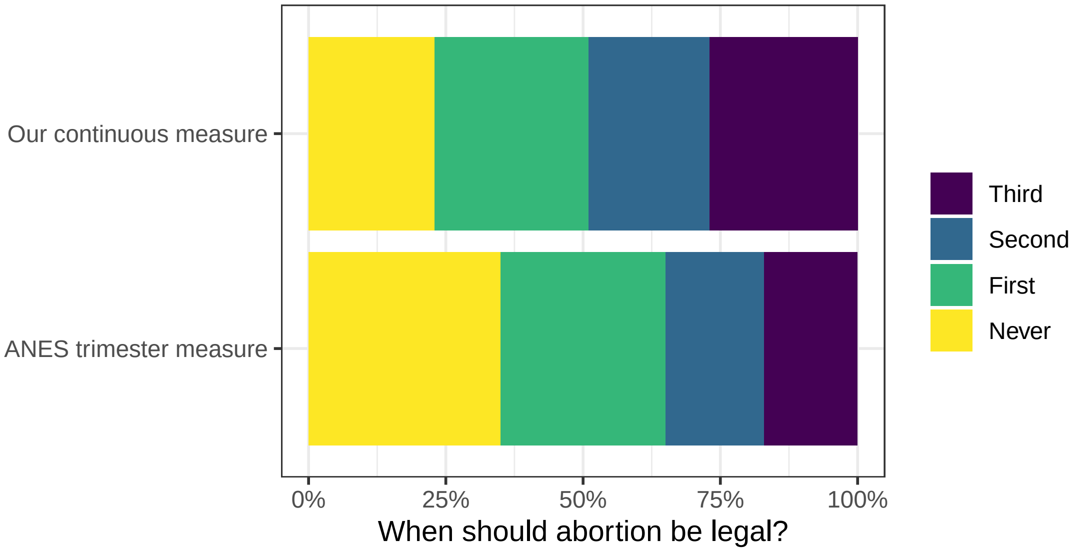

Figure S4: Validating our open-ended measure of gestational age policy preferences against ANES's trimester-based measure. Our data comes from the 3 pooled 2023 surveys. ANES data is from 2022 Pilot survey.

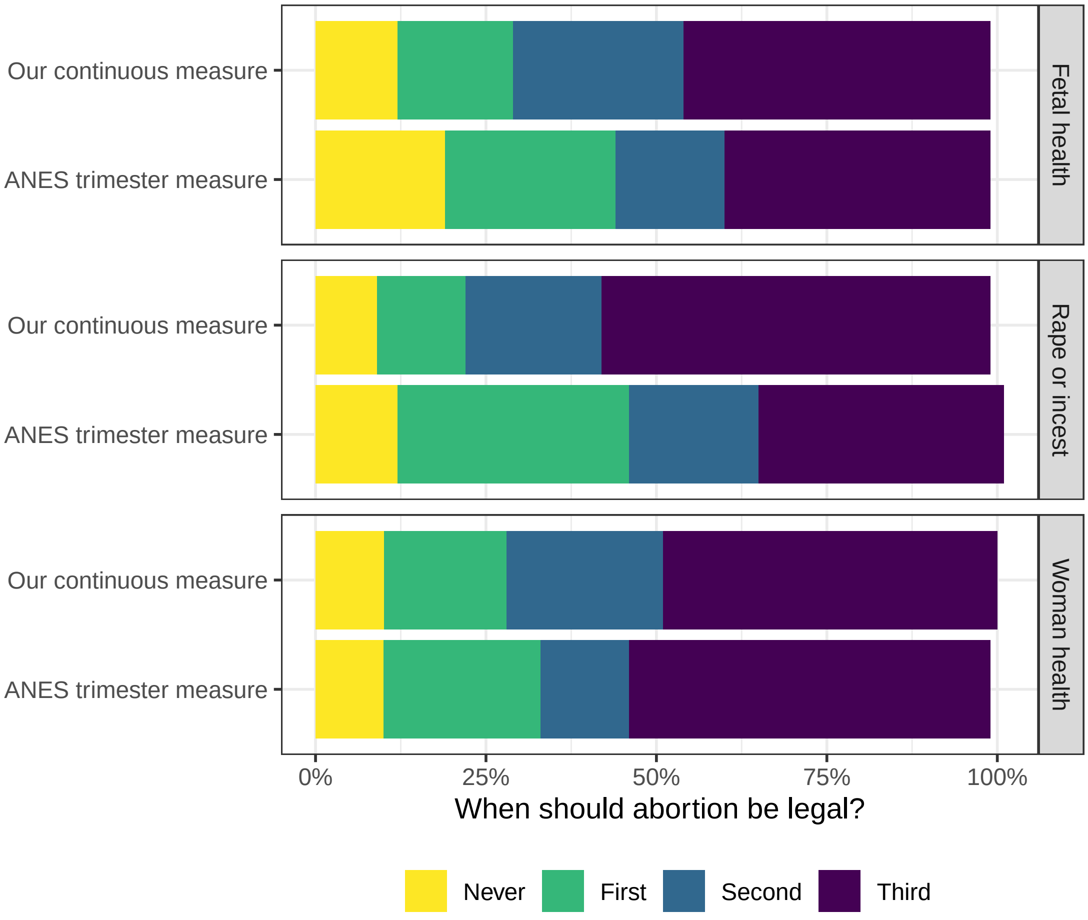

Figure S5: Validating our open-ended measure of gestational age policy preferences against ANES's trimester-based measure. Our data comes from the 2024 Lucid survey. ANES data is from 2022 Pilot survey.

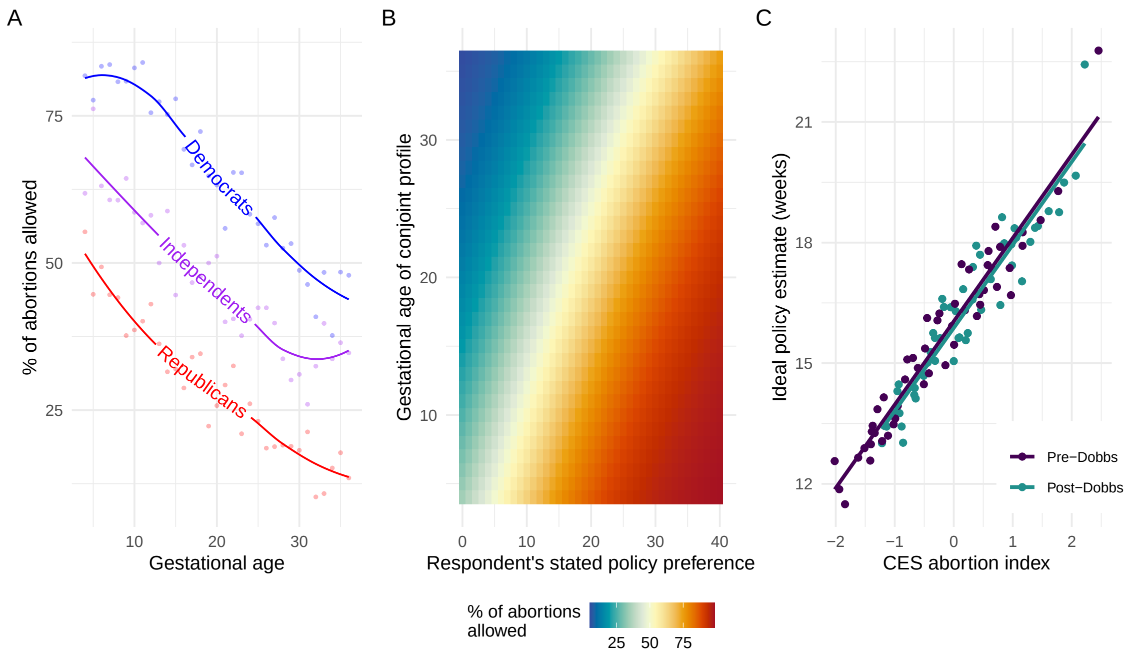

Figure S6: Validating our open-ended measure of gestational age policy preferences. Panel A displays the proportion of conjoint profiles for which abortion was allowed as a function of the gestational age and the respondents' partisanship. Points are means, the lines are loess curves. Panel B displays the proportion of profiles for which abortion was allowed as a function of gestational age and the respondents' stated policy preference. Panel C displays the state-level estimates of ideal abortion policy as a function of the CES's abortion index.

A.3.3 Experimental measurement approach

The Panel A of Figure S6 demonstrates the centrality of gestational age in American's attitudes towards legal abortion in hypothetical circumstances. The gradient of preferences with respect to gestational age is quite steep, dropping over 1% points per week. Moreover, gestational age also appears to trump group differences: even a near majority of Republicans appears to support the legality of abortion at the early weeks – when most abortions take place – and even the majority of Democrats are opposed to legalizing very late abortions. Panel B demonstrates that our open ended question picks up variation in these preferences quite accurately: respondents whose stated policy preference are very conservative (around 0 weeks) reject a large majority of profiles. Conversely, more liberal respondents (stated preference closer to 40 weeks) allow the abortion for most profiles. Finally, Panel C contrasts our state-level preferences based on the open-ended question with an index based on the CES, demonstrating that the two measures are strongly correlated (Pearson's rs > 0.94).

B Modeling and validation

Here is the R code of our main multilevel model:

```
ordbetareg(abort_open ~ 1 + abortion_index + trump +
                      (1 | age_fem) +
                      (1 | edu) +
                      (1 | racecat) +
                      (1 | state_name) +
                      (1 | region),
           data = data,
           backend = "cmdstanr",
           adapt_delta = 0.95,
           chains = 4,
           cores = 4)
```

Figure S7 displays the posterior predictive check. This is a visual diagnostic tool in the Bayesian analytic workflow intended to verify that our models can regenerate the data on which they were modeled.[4] The ordbetareg package in R offers the approach described below. On Panel A, we see that the model is very accurately back-predicts the proportion of respondents with a preference of 0 weeks, 40 weeks, or in between. Meanwhile, on Panel B) we see that our model picks up well on the overall distribution of the preferences between 0 and 40, but it is insensitive to local peaks at round numbers. Overall, we conclude that our model fits the data well.

[4]Gabry, J. , Simpson, D. , Vehtari, A. , Betancourt, M. and Gelman, A. (2019), Visualization in Bayesian workflow. J. R. Stat. Soc. A, 182: 389-402. doi:10.1111/rssa.12378.

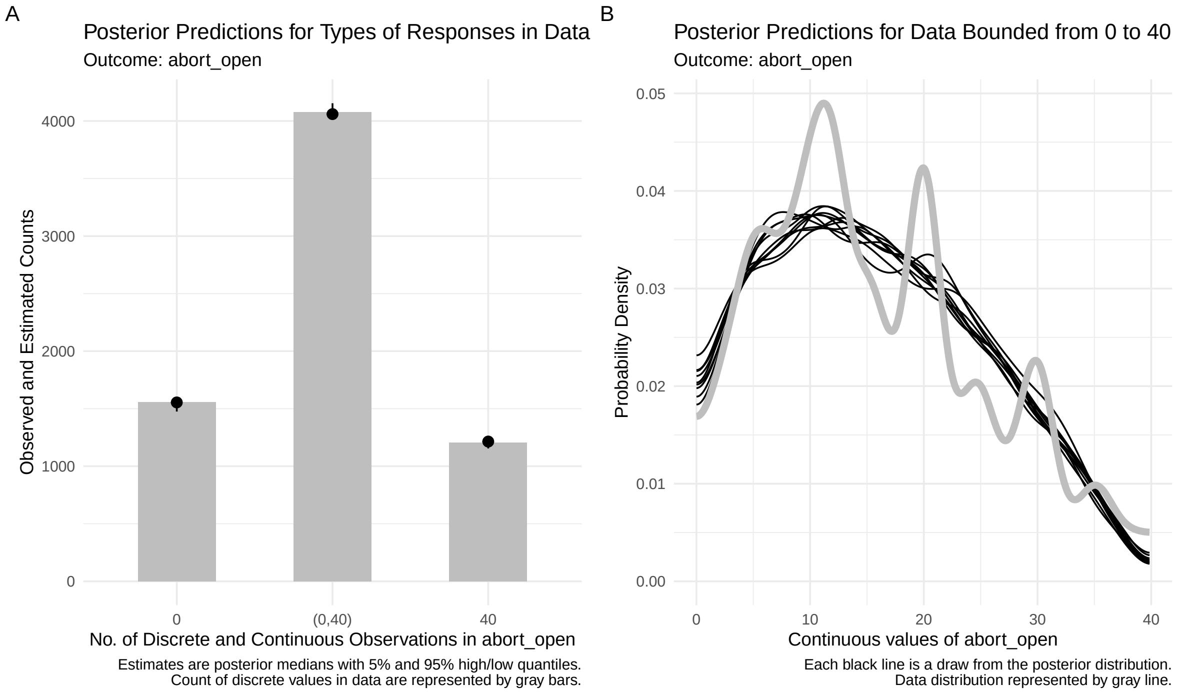

Figure S7: Posterior predictive checks for our ordered-beta regression model. Panel A displays observed and estimated levels of the 3 types of responses (preference at 0, at 40, or in between). Panel B displays the observed and predicted distribution for the latter type.

C Responsiveness estimates

The tables below describe the OLS regression models used to quantify policy responsiveness in the paper all. Each table reports the same model twice, first without any weights, and second using state-level population size as a weight. We report the latter as our main findings in the main text, although as it is seen the results are substantively similar for both. Tables S5 and S6 regress state policies on public preferences before and after the *Dobbs* decision, respectively. Table S7 quantifies the change in responsiveness between the two periods by including an interaction between preference and period. The estimates for the constants (intercepts) are substantively meaningless in all models and quantify the expected policy for a hypothetical states where the preferred gestational age limit is 0 weeks.

Table S5: Responsiveness Pre-Dobbs

	Dependent variable:	
	State policy Pre-Dobbs	
	(1)	(2)
Preference	1.56***	1.16***
	(1.01, 2.11)	(0.58, 1.74)
Constant	1.65	6.24
	(−6.98, 10.28)	(−2.99, 15.46)
Weights	No	Yes
R^2	0.31	0.18
Residual Std. Error (df = 49)	5.28	0.58
Note:	*p<0.1; **p<0.05; ***p<0.01	

Table S6: Responsiveness Post-Dobbs

	Dependent variable:	
	State policy Post-Dobbs	
	(1)	(2)
Preference	5.56***	6.07***
	(4.63, 6.49)	(4.78, 7.36)
Constant	−73.33***	−82.54***
	(−88.65, −58.01)	(−103.89, −61.19)
Weights	No	Yes
R^2	0.66	0.55
Residual Std. Error (df = 49)	7.88	1.12
Note:		*p<0.1; **p<0.05; ***p<0.01

Table S7: Change in Responsiveness

	Dependent variable:	
	State policy	
	(1)	(2)
Preference	1.56***	1.16**
	(0.86, 2.25)	(0.28, 2.05)
Period (Post)	−74.97***	−88.78***
	(−92.01, −57.94)	(−110.88, −66.67)
Preference x Period (Post)	4.01***	4.91***
	(2.95, 5.06)	(3.55, 6.27)
Constant	1.65	6.24
	(−9.31, 12.61)	(−7.84, 20.32)
Weights	No	Yes
R^2	0.65	0.57
Residual Std. Error (df = 98)	6.71	0.89
Note:		*p<0.1; **p<0.05; ***p<0.01

D CES and abortion policies

We use a state-level abortion policy estimate using survey questions from the 2020 and 2020 Cooperative Election Study (CES). Specifically, we average and z-score the factor scores for each respondent from all six questions about abortion policy that were asked both in 2020 and 2022. Table S8 displays the items and the factor loadings for each. Higher values denote support for more liberal abortion policies.

To calculate state-year averages, we relied on a simple multilevel regression model with a factor for year as the only predictor, while including varying intercepts and varying slopes for states. This model has a better fit than a model without year as predictor, or without varying slopes.

Table S8: Factor loadings from CES abortion policy data

item	loading
Always allow a woman to obtain an abortion as a matter of choice	0.70
Permit abortion only if rape, incest, or wmn's life in danger	0.48
Prohibit all abortions after the 20th week of pregnancy	0.62
Allow employers to decline coverage of abortions in insurance plans	0.77
Prohibit the expenditure of [federal] funds ... for any abortion	0.80
Make abortions illegal in all circumstances	0.34

Figure S8 displays the state-level average support for abortion policy in 2020 versus 2022. Between these two waves of the CES, attitudes became slightly more liberal on average.

Figures S9 and S10 replicate our main analyses of policy responsiveness (see Figure **??** of the manuscript), using alternative measures of abortion policy preferences relying on the Cooperative Election Study (CES). In each case we see a strengthening of 'responsiveness' post-Dobbs.

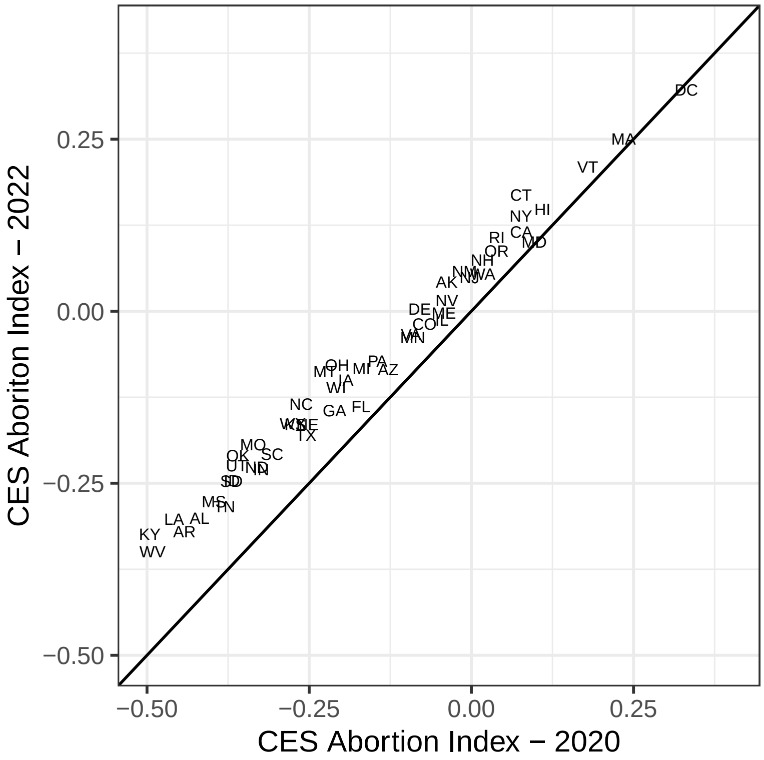

Figure S8: State-level support for abortion slightly increased between 2020 and 2022. Each text label denotes a state average relying on the CES abortion index. The horizontal line denotes the diagonal of no change. The index is scaled to respondent-level standard deviations.

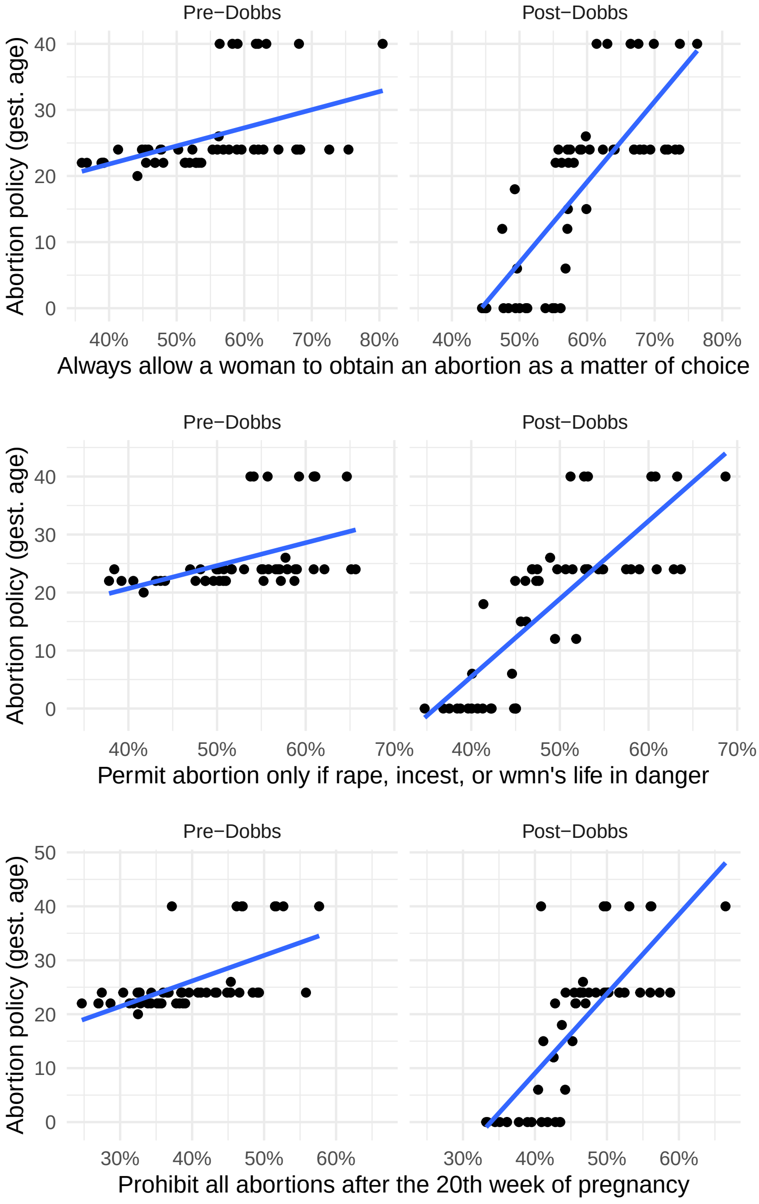

Figure S9: Abortion policy as a function of public sentiment. Relying on CES data - Part 1. Dots denote survey weighted average support or rejection of the item in each state (x-axis). All items are scaled such that high values indicate more liberal average views. Lines denote OLS regression lines.

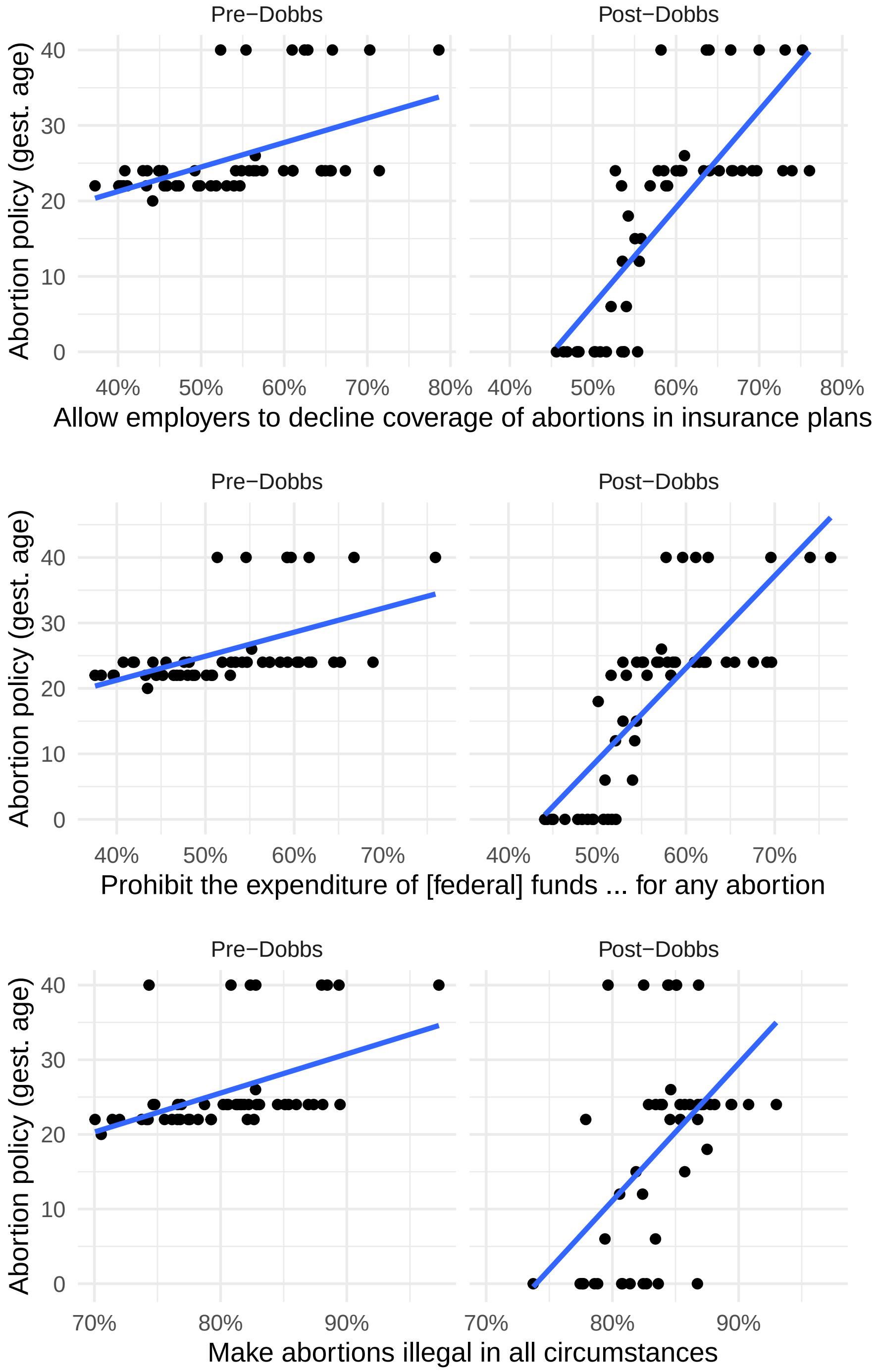

Figure S10: Abortion policy as a function of public sentiment. Relying on CES data - Part 2 Dots denote survey weighted average support or rejection of the item in each state (x-axis). All items are scaled such that high values indicate more liberal average views. Lines denote OLS regression lines.

E Top- and bottom-coding gestational age

Because it takes 40 weeks to carry a pregnancy to term, we offered the theoretical range from 0 to 40 weeks to indicate abortion preferences in our open-ended question. However, admittedly, an overwhelming majority of abortions happen at a much narrower range in gestational age. On the one hand, women often do not realize that they are pregnant in the first four weeks. On the other hand, very few abortions are performed after 24 weeks once the fetus is considered viable (**?**). To test the robustness of our findings, we replicate our analysis, top- and bottom-coding responses to restrict the abortion preference measure to the range between 4 and 24 weeks. Table S9 and Figure S11 demonstrate that our results remain substantively similar to our main analyses.

Table S9: Policy responsiveness - top and bottom coding

	Pre-Dobbs	Post-Dobbs	Difference
Policy	23.36	17.08	-6.28
Preference	18.74	19.61	0.87
	(17.44, 20.06)	(18.38, 20.86)	(0.44, 1.33)
Liberal Bias	4.62	-2.53	-7.14
	(3.30, 5.92)	(-3.78, -1.30)	(-7.61, -6.71)
Absolute Bias	4.63	6.18	1.55
	(3.33, 5.93)	(4.97, 7.39)	(0.27, 2.81)
Responsiveness	0.3	3.8	3.49
	(0.22, 0.39)	(3.08, 4.51)	(2.83, 4.15)

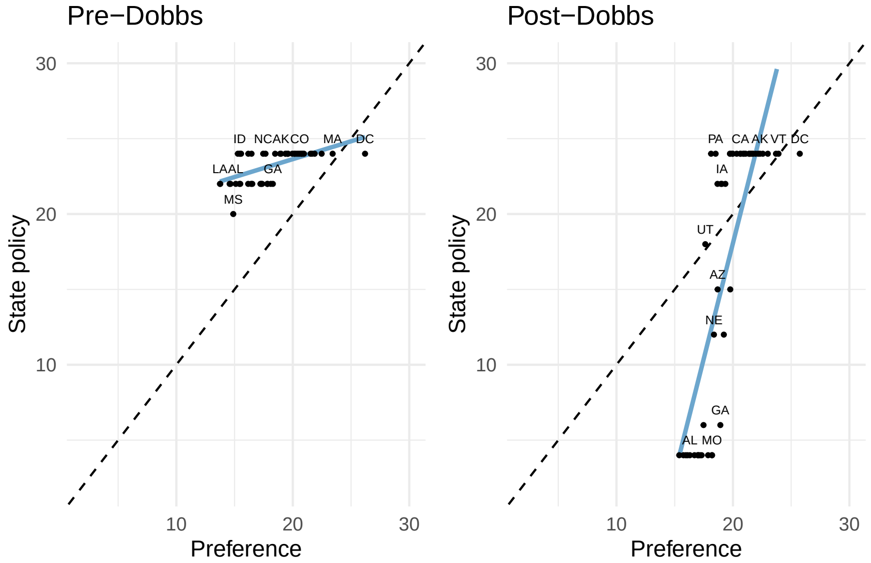

Figure S11: Policy responsiveness and bias in abortion laws after top and bottom-coding abortion preferences. Points denote states. The blue lines denote OLS regression lines.

F Excluding New Hampshire

Table S10: Policy responsiveness - top and bottom coding

variable	Pre-Dobbs	Post-Dobbs	Difference
Policy	24.55	17.29	-7.27
Preference	15.49	16.45	0.95
	(14.31, 16.70)	(15.36, 17.55)	(0.48, 1.44)
Liberal Bias	9.06	0.84	-8.22
	(7.85, 10.24)	(-0.27, 1.92)	(-8.70, -7.75)
Absolute Bias	9.06	9.19	0.13
	(7.85, 10.24)	(8.10, 10.28)	(-0.99, 1.28)
Responsiveness	1.06	5.98	4.92
	(0.51, 1.61)	(4.66, 7.3)	(3.58, 6.26)

G Benchmarking experiment

Admittedly, many of our respondents have no detailed knowledge about fetal development, which creates a risk that we cannot to take literally the answers people give. To address this concern, we collected additional data where respondents answered questions about gestational age preferences with and without anchors. Specifically, we ran a survey experiment following best advice from Ansolabehere and colleagues[5] either giving only the same a minimal benchmark to respondents as we did in our main study ("a typical full-term pregnancy lasts 40 weeks"), or giving 4 more benchmarks commonly mentioned in debates about abortion. The box below displays our question wording with the treatment highlighted in **bold**. The order of the first three items were randomized with the option "none of these circumstances apply" always coming last.

We fielded this experiment as part of an omnibus survey fielded on Lucid Theorem to a sample of 2683 US American respondents in early November, 2024 (the days before the elections). 75% of our sample saw the version with the benchmark and the remaining 25% got the control.

[5] Ansolabehere, S., Meredith, M., & Snowberg, E. (2013). Asking about numbers: Why and how. Political Analysis, 21(1), 48-69.

Now we would like to ask you about the abortion laws in your state. In your opinion, what number of weeks should be the LATEST point when a woman should be allowed to have an abortion in your state?
As a reminder, a typical full-term pregnancy lasts 40 weeks. **The heart starts beating around week 5-6. The fetus starts to make active movements around week 14-16. If born prematurely, a baby in the US has around 50% chance of survival at week 24, and 95% chance from week 28.**
If you think women should not be allowed to have an abortion please enter 0, below. If you think women should be allowed to have an abortion at any point of the pregnancy, please enter 40 below.
Please consider each of the following circumstances and enter a number in between 0 and 40.

- The health of the woman is endangered:
- There is a risk of serious birth defects in the fetus:
- The woman was victim of rape or incest:
- None of these circumstances apply:

We report an OLS regression model regressing abortion preferences on an indicator which switches on if the respondent saw the version of the question with the benchmarks. Figure S12 displays the distributions of these variables across the two conditions, highlighting large overlaps. Table S11 demonstrates that all experimental effects are very close to 0. These results also demonstrate that there are marked differences between the average gestational age threshold when special circumstances do or do not apply. Specifically, for rape or incest, or health risks to the woman or the fetus the average respondent prefers a limit above viability (24 weeks, 27 weeks, or 24 weeks, respectively). Meanwhile, in this framework, the average threshold without special circumstances is set at just 10 weeks. Admittedly, this difference may be exacerbated by unforeseen contrasts effects, wherein respondents gave more conservative answers for the last category, to stay consistent with their prior answers. Yet, the average threshold between pregnancies with and without special circumstances is large even if we contract our novel results

to the 2023 Lucid survey, where the average gestational age threshold was 16.5 weeks.

Table S11: Experimentally introducing gestational age milestones as anchors does not affect abortion policy preferences

	Dependent variable:			
	Rape or Incest	Woman's health	Fetus's health	None of these
	(1)	(2)	(3)	(4)
Anchor	0.4	−0.4	−0.7	0.1
	(−0.9, 1.7)	(−1.6, 0.9)	(−1.9, 0.6)	(−1.1, 1.3)
Constant	24.3***	27.1***	23.8***	9.7***
	(23.2, 25.4)	(26.1, 28.2)	(22.7, 24.9)	(8.7, 10.8)
Observations	2,682	2,682	2,682	2,682
Adjusted R^2	0.00	0.00	0.00	0.00
Note:			*p<0.1; **p<0.05; ***p<0.01	

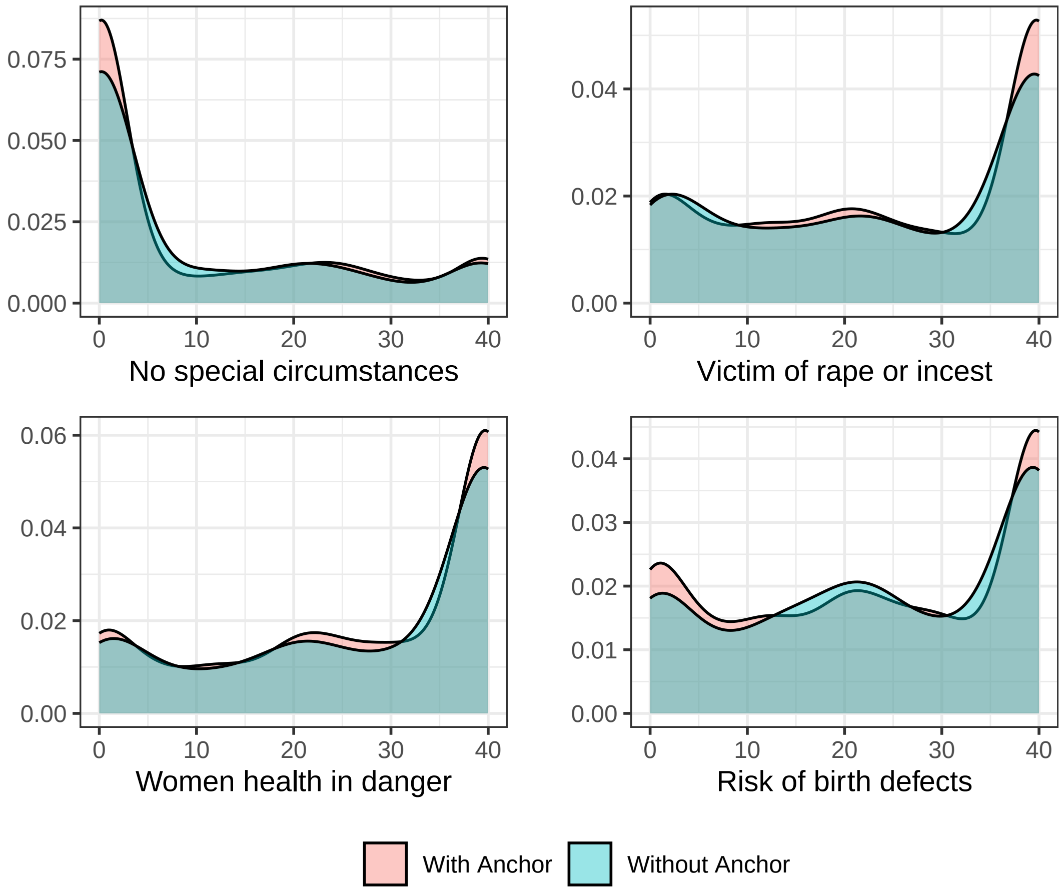

Figure S12: Density distributions with and without the anchors completely overlap. We see no evidence that presenting respondents information about several gestational milestones changes their gestational age policy preferences.

H MRP estimates versus state polls

Following the advice of an anonymous reviewer we also compare our state level estimates of abortion preferences to a set of available state-polls, fielded in Tennessee, Pennsylvania, North Carolina and Missouri. Because our estimates are on a cardinal scale – i.e. the ideal abortion policy observed at the individual level – we make use of our multi-level model to construct estimates of support for policy changes corresponding to the state-level polls. Specifically, we follow the procedure below:

1. Using our main regression model of gestational age limit preferences, we simulate 2000 hypothetical responses for each census cell.
2. For each simulated respondent, we construct an indicator that takes the value of 1, if the respondent's ideal point is below the specific threshold used in the given state poll. For instance, our benchmark Tennessee state poll asked respondents if they think "think abortion should be illegal after 15 weeks except in cases of rape, incest, or to save the life of the mother". Our approach assumes that all simulated Tennesseans whose ideal point equals or is below 15 weeks would answer "yes" to this question, and all those whose ideal point is above 15 weeks would answer "no".
3. Relying on census weights, we calculate the weighted average of our simulated respondents who would support the given abortion ban specified in the survey question in the given state.
4. We compare the estimated support with the aggregate results in the survey.

As shown in Table S12 our estimates are broadly consistent with the state level polls, indicating some face validity of our estimates. Note however, that this validation requires many untestable assumptions. First, some of the questions included in the state polls do not correspond to specific policy changes. Second, the polls also include a "Don't know option". Third, the poll results themselves also reflect uncertainty resulting from sampling error and possible question wording effects. Thus our preferred interpretation is that these state polls provide some face validity to our estimates rather then validate them in some exact way.

Table S12: Proportion of respondents supporting an abortion ban at given gestational age

	Gest Age Limit	Poll Estimate	MRP estimate	95%CI Lwr	Upr
Missouri	8 weeks	0.36	0.4	0.25	0.56
Pennsylvania	15 weeks	0.44	0.56	0.4	0.7
North Carolina	12 weeks	0.51	0.46	0.33	0.6
Missouri	15 weeks	0.53	0.56	0.39	0.71
Tennessee	15 weeks	0.72	0.6	0.43	0.74
